# Single-Pulse TMS over the Parietal Cortex Does Not Impair Sensorimotor Perturbation-Induced Changes in Motor Commands

**DOI:** 10.1523/ENEURO.0209-19.2020

**Published:** 2020-03-11

**Authors:** Félix-Antoine Savoie, Lauranne Dallaire-Jean, François Thénault, Kevin Whittingstall, Pierre-Michel Bernier

**Affiliations:** 1 Département de médecine nucléaire et radiobiologie, Faculté de médecine et des sciences de la santé, Université de Sherbrooke, Sherbrooke, Québec J1H 5N4, Canada; 2 Département de kinanthropologie, Faculté des sciences de l’activité physique, Université de Sherbrooke, Sherbrooke, Québec J1K 2R1, Canada; 3 Centre de Recherche du Centre Hospitalier Universitaire de Sherbrooke (CHUS), Université de Sherbrooke, Sherbrooke, Québec J1H 5N4, Canada

**Keywords:** adaptation, parietal, sensorimotor adaptation, TMS, visuomotor rotation

## Abstract

Intermittent exposure to a sensorimotor perturbation, such as a visuomotor rotation, is known to cause a directional bias on the subsequent movement that opposes the previously experienced perturbation. To date, it is unclear whether the parietal cortex is causally involved in this postperturbation movement bias. In a recent electroencephalogram study, Savoie et al. (2018) observed increased parietal activity in response to an intermittent visuomotor perturbation, raising the possibility that the parietal cortex could subserve this change in motor behavior. The goal of the present study was to causally test this hypothesis. Human participants (*N* = 28) reached toward one of two visual targets located on either side of a fixation point, while being pseudorandomly submitted to a visuomotor rotation. On half of all rotation trials, single-pulse transcranial magnetic stimulation (TMS) was applied over the right (*N* = 14) or left (*N* = 14) parietal cortex 150 ms after visual feedback provision. To determine whether TMS influenced the postperturbation bias, reach direction was compared on trials that followed rotation with (RS + 1) and without (R + 1) TMS. It was hypothesized that interfering with parietal activity would reduce the movement bias following rotated trials. Results revealed a significant and robust postrotation directional bias compared with both rotation and null rotation trials. Contrary to our hypothesis, however, neither left nor right parietal stimulation significantly impacted the postrotation bias. These data suggest that the parietal areas targeted here may not be critical for perturbation-induced motor output changes to emerge.

## Significance Statement

The parietal cortex is known to contribute to sensorimotor adaptation. Despite this, it remains unclear whether it contributes to the automatic and nonstrategic changes in motor behavior that occur following exposure to a sensorimotor perturbation. In the present study, we show that disrupting parietal activity in the vicinity of the angular gyrus using single-pulse transcranial magnetic stimulation (TMS) shortly after exposure to a visuomotor perturbation does not impact reach direction on a subsequent movement. Although these results must be interpreted in light of the spatiotemporal characteristics of the TMS protocol used, they suggest that the aforementioned parietal areas may not be critical for the emergence of the motor output adjustments that take place in response to a visuomotor perturbation.

## Introduction

An important aspect of motor learning concerns the adaptation of existing motor skills to various perturbations (i.e., sensorimotor adaptation; Krakauer et al., 2019). At least two processes are thought to contribute to sensorimotor adaptation. The explicit process, mainly driven by performance errors (i.e., the discrepancy between the movement goal and outcome), is thought to reflect a strategic reaiming to counter the perturbation (Taylor et al., 2014; McDougle et al., 2015). The implicit process, which is mainly driven by sensory prediction errors (SPE; i.e., the mismatch between the predicted and actual sensory consequences of a movement), is thought to reflect the updating of an internal forward model that allows the motor system to accurately predict the sensory consequences of movement (Taylor et al., 2014; McDougle et al., 2015). In the field of sensorimotor learning, an important endeavor has been to identify the neural correlates of both explicit and implicit adaptation.

One of the most frequently reported neural correlates of sensorimotor adaptation is the parietal cortex (Inoue et al., 1997, 2000; Shadmehr and Holcomb, 1997; Ghilardi et al., 2000; Krakauer et al., 2004; Graydon et al., 2005; Seidler et al., 2006; Girgenrath et al., 2008; Seidler and Noll, 2008; Luauté et al., 2009; Werner et al., 2014). Given evidence that disrupted parietal activity impairs one’s ability to compensate for a sensorimotor perturbation without necessarily impacting postadaptation aftereffects (Della-Maggiore et al., 2004; Pisella et al., 2004; Panico et al., 2018), it has been suggested that this area may be selectively involved in explicit adaptation. For instance, Panico et al. (2018) showed that applying either anodal or cathodal transcranial direct current stimulation (tDCS) over the parietal cortex impairs the ability to correct for target errors during prism adaptation without impacting postadaptation aftereffects. However, some studies have shown that parietal lesions impair both adaptation and subsequent aftereffects, suggesting that the parietal cortex may play a role in implicit adaptation as well (Newport et al., 2006; Newport and Jackson, 2006; Mutha et al., 2011a,b). While the above-mentioned studies indicate that the parietal cortex is involved in explicit adaptation, its involvement in implicit adaptation is more ambiguous.

One way to probe for implicit adaptation is to expose individuals to an intermittent or randomly changing perturbation and to investigate the directional movement bias that occurs on the following trial (Diedrichsen et al., 2005; Galea et al., 2015; Torrecillos et al., 2015; Tan et al., 2016; Savoie et al., 2018). Indeed, this bias has been suggested to be automatic and nonstrategic (Donchin et al., 2003; Galea et al., 2015), which arguably make it a valid proxy for implicit adaptation. As a matter of fact, the magnitude of postperturbation movement biases (Diedrichsen et al., 2005; Galea et al., 2015) is typically similar to the learning rate reported for implicit adaptation (Smith et al., 2006; McDougle et al., 2015). Interestingly, a recent electroencephalography (EEG) study has provided evidence that the parietal cortex may be involved in the directional reaching bias that emerges following single-trial exposure to a visuomotor rotation (Savoie et al., 2018). In that study, participants made reaching movements toward a visual target while being pseudorandomly exposed to a 45° visuomotor rotation (approximately every three trials). Importantly, participants knew which trials would and would not be perturbed and were given an aiming strategy to successfully counter the rotation on perturbed trials. Despite this, participants showed a significant reaching bias in the direction opposite to the perturbation following rotated trials. Critically, when rotated trials were compared with control trials matched for motor output, sensory input, and performance errors, a phasic parietal response was observed 140–260 ms after movement onset. Given that the only major difference between the rotated and control trials was the presence of an SPE, the authors speculated that this parietal response reflected SPE processing and, therefore, the engagement of implicit adaptation mechanisms. Although reasonable, this interpretation was speculative, as the authors did not assess a causal relationship between the visuomotor rotation-induced parietal activity and subsequent directional reaching bias.

In light of the above, the goal of the present study was to test a causal relationship between the parietal responses observed by Savoie et al. (2018) and the movement bias incurred by single-trial exposure to a visuomotor rotation. To do so, single-pulse transcranial magnetic stimulation (TMS) was used to disrupt either left or right parietal activity as participants were intermittently exposed to a 45° visuomotor rotation. The spatiotemporal parameters of the TMS protocol were specifically chosen to disrupt the visuomotor rotation-induced parietal activity reported by Savoie et al. (2018). It was hypothesized that disrupting parietal activity at the putative moment of SPE processing would reduce the postrotation bias compared with a control (i.e., no TMS) condition.

## Materials and Methods

### Participants

Twenty-eight right-handed and neurologically healthy university students, who were randomly divided into two equal groups [left parietal stimulation group (P3): 9 females, 24 ± 3 years old (mean ± SD); right parietal stimulation group (P4): 10 females, 24 ± 4 years old; see TMS protocol], took part in this study. Based on self-report, all had normal or corrected-to-normal vision. Each participant completed the questionnaire by Lefaucheur et al. (2011) prior to participation to determine whether they were eligible for TMS. All procedures were approved by the Université de Sherbrooke institutional review board and ethics committee and fully explained to participants prior to obtaining their informed and written consent.

### Experimental setup

The experimental setup consisted of a steel frame mounted on a tabletop, which supported a 23 inch computer monitor (model VH238H, ASUS) that projected visual stimuli onto a semisilvered mirror positioned in front of participants. The monitor (resolution: 1920 × 10^80^; refresh rate: 75 Hz) was mounted face down 29 cm above the semisilvered mirror, which was positioned 29 cm above the table surface. Hand movements were recorded by way of a custom-built manipulandum composed of two lightweight metal rods, which lay on the table surface below the mirror. To move the manipulandum, participants used a short steel handle located at its mobile end. Two potentiometers, located at the hinges of the manipulandum, allowed for the recording of movement-induced changes in rod angle at 100 Hz, from which planar hand displacements were determined. To minimize friction between the manipulandum and table surface, a smooth plastic sheet was fixed to the table and felt pads were secured beneath the hinges of the manipulandum. This setup allowed participants to view the visual stimuli in the same plane as their hand. Moreover, because all experiments were conducted in the dark, the semisilvered mirror prevented participants from seeing their hand during the experiment.

### Experimental task

#### Overview

**Figure 1.**
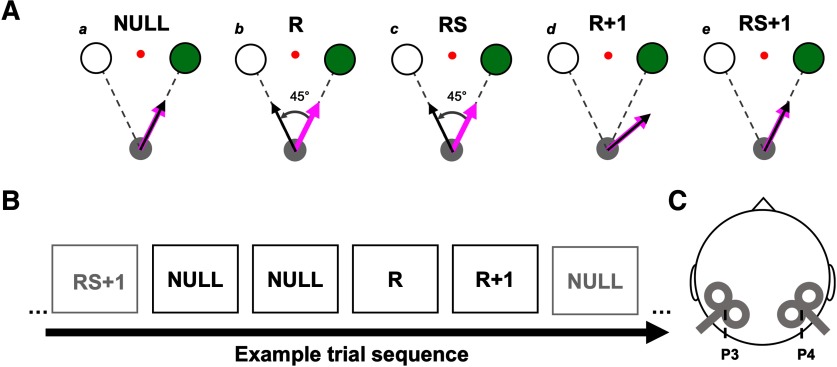
Study design. ***A***, Experimental conditions NULL trials (***a***), rotation trials (*R*; ***b***), RS trials (***c***), R trials (***d***), and RS + 1 trials (***e***). In ***a–e***, the target to reach is represented in green, the magenta arrow represents hand direction, the black arrow represents the cursor direction and the red circle represents the fixation point. It was hypothesized that parietal TMS would attenuate postrotation directional bias (i.e., trial-by-trial changes in motor behavior) on RS + 1 compared with R + 1 trials (see ***d*** vs ***e***). ***B***, Example trial sequence. At least two NULL trials separated each R + 1/RS + 1 trial from the following R/RS trial. ***C***, Depiction of stimulation sites P3 and P4 and coil positioning.

On each trial, participants were required to bring a virtual cursor (white circle, diameter: 0.85 cm) from a start base to one of two visual targets by reaching toward it with their right arm (Fig. 1*Aa*). The start base (Fig. 1*Aa*, gray circle; diameter: 1.06 cm) was positioned 30 cm in front of participants and aligned to their midline, whereas the two targets (Fig. 1*Aa*, circles with black outline; diameter: 2.12 cm) were located 12 cm away from the start base and on either side (22.5°) of participants’ midline. To control for gaze position during the experiment, participants were asked to maintain their eyes on a fixation point (Fig. 1*Aa*, red circle; diameter: 0.27 cm), which was located 12 cm in front of the start base along the midline (i.e., between the two targets). All visual landmarks (i.e., start base, targets and fixation point) were visible throughout the experiment.

#### Trial timeline

All trials were initiated when participants actively brought the cursor within the start base. If this position was maintained for 2 s, one of the two visual targets turned green, instructing participants to initiate their reach toward that target (i.e., go-cue). During the reaching portion of all trials, participants were provided with full visual feedback of the cursor (i.e., closed-loop feedback). Participants were instructed to land the cursor within the cued target and complete their reaching movement in <200 ms to minimize online corrections. Although participants were not pressed to react as quickly as possible [i.e., this was not a reaction time (RT) task], they were encouraged to react shortly after the go-cue. If participants landed their cursor within the target and their movement time (MT) was <200 ms, the target exploded to indicate a successful trial. If participants landed within the target but movement time was ≥200 ms, the target was colored in blue to inform participants that they had moved too slowly. Finally, if participants failed to land on the target, it turned red, indicating that they had missed the target. Importantly, participants were told that the goal of the task was to make the target explode on as many trials as possible. At movement end, the virtual cursor was extinguished and a short text indicating movement time (e.g., 189 ms) appeared 1 cm above the fixation point. This provided participants with feedback concerning their movement time and also informed them that the trial was complete and that they should bring their hand back to the start base to initiate the next one. Given that the cursor was still not visible at this point, a metal “V”-shaped dock was fixed to the workspace surface so that participants could simply bring their hand back toward their midline until they hit the dock, after which they could slide the manipulandum to the start base. The cursor was made visible again, and performance feedback extinguished, only when the next trial was initiated.

#### Experimental timeline and conditions

After a familiarization period (≥50 trials, depending on how comfortable the participant was with the task), participants completed 600 experimental trials. Three hundred sixty of these were null rotation trials, in which participants were provided with veridical visual feedback of their hand (NULL; Fig. 1*Aa*). Amid these NULL trials were embedded 60 rotation (R) and 60 rotation + stimulation (RS) trials, in which a 45° visuomotor rotation was imposed on the cursor (Fig. 1*Ab*,*Ac*). On left target R/RS trials, the rotation was counterclockwise (i.e., positive), whereas on right target R/RS trials, the rotation was clockwise (i.e., negative). R and RS trials were identical except that in RS trials, participants received a TMS pulse over the left or right parietal cortex depending on the group they belonged to (see TMS protocol). R and RS trials were respectively followed by R + 1 and RS + 1 trials, which were free of visuomotor rotation and always directed toward the same target as the preceding R or RS trial (Fig. 1*Ad*,*Ae*). The purpose of R + 1 and RS + 1 trials was to probe for the directional reaching bias following exposure to the visuomotor rotation. At least two NULL trials, pseudorandomly directed toward the right or left target, separated each R/R + 1 or RS/RS + 1 pair to wash out any adaptation that might take place. An example trial sequence is depicted in Figure 1*B*. In each condition, trials were evenly split between left and right target trials.

During the experiment, participants were not aware of which trials would be rotated. Importantly, they were told to aim directly toward the cued target (i.e., the one that turned green) on every trial, regardless of what the cursor did. Moreover, participants were told that trying to guess and implement a strategy to counter the visuomotor rotation was counterproductive, as these trials would not contribute toward their final score (i.e., number of hits). Additionally, to ensure that participants made a genuine effort to directly reach toward the target on postrotation trials, they were told that the trial immediately following a rotated trial would never be submitted to a visuomotor rotation. Given these instructions, it was reasoned that any directional reaching bias on R + 1 and RS + 1 trials would be free of any aiming strategies (i.e., it would be implicitly driven). None of the participants were aware of the research hypothesis prior to taking part in the experiment.

### Movement-related data recording and analysis

All visual stimuli were presented using functions from the Psychtoolbox (Brainard, 1997; Pelli, 1997), which was run using MATLAB (version 2014a, MathWorks). Movement-related data, which were recorded with the potentiometers of the manipulandum, were analyzed offline using custom MATLAB code. For all trials, movement initiation was defined as the first time sample when the position of the hand was recorded outside the start base following the go-cue, whereas movement termination was defined as the first time sample when hand velocity was <1 pixel/s after movement initiation. RT was calculated as the time between the go-cue and movement initiation, whereas MT was calculated as the time between movement initiation and termination. Although RT and MT were not of special interest in this study, they were analyzed to assess whether motor behavior was similar with and without TMS. Reach angle at peak velocity, which was used to assess the postrotation bias, was defined as the angular difference between the target vector (i.e., start base to cued target) and hand vector (i.e., start base to hand position) at peak velocity. Radial target error, which was used to identify potential outlier trials prior to data analysis (see Movement-related trial rejection), was defined as the distance between the position of the hand and the target center at movement termination. Since radial target errors, as defined above, are insensitive to the direction of error, they were further broken down into mediolateral (Final^x^) and anteroposterior (Final^y^) components and used as additional variables to estimate reach direction throughout the experiment. Given that premovement parietal stimulation has previously been reported to increase movement variability (Vesia et al., 2008, 2010), we also determined reach angle variability at peak velocity, as well as Final^x^ and Final^y^ variability, and compared these variables across conditions.

### Movement-related trial rejection

Visual inspection of all trials revealed that four participants each completed one reaching movement to the right target on a left target trial (one in RS + 1, three in R + 1). These trials were removed from the analyses given that participants either voluntarily or involuntarily did not follow the instructions. To further prevent outlier trials from affecting movement-related outcomes, thresholds were established to reject trials based on RT (≤120 ms, 11 total trials identified) and MT (>300 ms, 226 total trials identified). These thresholds were chosen because (1) RTs <120 ms were deemed too quick to be valid (Haith et al., 2016) and (2) MTs >300 ms were deemed too long given that the goal of the task was to hit the target within 200 ms (see Trial timeline). On average, this led to the rejection of 8 ± 13 trials (range, 0–53) per participant (left target: NULL, 3 ± 5, range, 0–20; R, 1 ± 1, range, 0–6; RS, 1 ± 3, range, 0–10; R + 1, 0 ± 0, range, 0–2; RS + 1, 2 ± 3, range, 0–13; right target: NULL, 0 ± 0, range, 0–1; R, 0 ± 1, range, 0–5; RS, 0 ± 1, range, 0–3; R + 1, 0 ± 1, range, 0–4; RS + 1, 0 ± 1, range, 0–4).

### TMS protocol

In a previous EEG study, Savoie et al. (2018) identified parietal responses to visuomotor SPEs in healthy human participants. This activity was apparent between 140 and 260 ms after movement onset with online visual feedback, peaking at electrode P4 of the extended 10–20 electrode coordinate system. In the present study, we tested whether these parietal responses were related to implicit adaptation by interfering with the parietal cortex using single-pulse TMS. Single-pulse TMS was used because it enabled the transient disruption of neural activity at the presumed moment of SPE processing (i.e., ∼140–260 ms after feedback provision), while sparing task-related processes taking place in other time windows. Neither offline repetitive TMS nor tDCS could have permitted this, as the effects of these stimulation techniques are known to linger for several minutes after stimulation (Nitsche et al., 2008; Lefaucheur et al., 2011). On RS trials (see Experimental timeline and conditions), single-pulse TMS was delivered using a MagStim 200 monophasic stimulator (MagStim), using a 70-mm-diameter figure-of-eight coil positioned over either the right or left parietal cortex. In the P4 group (*N* = 14), the coil was centered over P4, whereas in the P3 group (*N* = 14), the coil was centered over P3. Rather than using an EEG cap to identify P3 and P4, an electrode-free rubberized swimming cap (Speedo) was used, allowing the coil to be positioned closer to the scalp during the experiment. Moreover, the rubberized cap provided better adherence with the coil, which minimized potential coil movements during the experiment. To identify P3 and P4, the 10–20 electrode coordinate system, as described by Oostenveld and Praamstra (2001), was mapped onto each participant’s head. First, the distance between nasion and inion (35.3 ± 1.7 cm across all participants) was determined, and felt markers were used to identify the 10% (FPz), 50% (Cz), 70% (Pz), and 90% (Oz) locations. Second, the distance between the right and left preauricular points (36.9 ± 1.4 cm across all participants) was determined and the 10% (T7), 50% (Cz), and 90% (T8) locations were identified. Importantly, special care was taken to ensure that the nasion–inion and preauricular measurements always intersected at their midway point (i.e., Cz). Third, the circumference of the head (passing through FPz, T7, Oz, and T8) was determined, and the 35% (P7) and 65% (P8) locations were identified with the felt marker. The midway point between P7 and Pz was taken as P3, whereas the midway point between Pz and P8 was taken as P4. Neither the nasion–inion nor the preauricular distances were significantly different between P3 and P4 groups (independent *t* tests: *t*_(26)_ = −0.26 and 0.83, *p *≥* *0.41). For both stimulation groups, the coil was positioned tangentially to the scalp with the handle pointing posterolaterally at a 45° angle (Fig. 1*C*; Vesia et al., 2010).

Although the acute effects of single-pulse TMS may only last between 5 and 40 ms (Sliwinska et al., 2014), TMS-EEG studies have shown that cortical activity is perturbed for up to 300 ms following the delivery of a single TMS pulse (Chung et al., 2015), with the first 100 ms likely relating to inhibitory cortical processes (Premoli et al., 2014; Chung et al., 2015). Considering this, as well as the latency of parietal responses to visuomotor SPE (i.e., 140–260 ms; Savoie et al., 2018), it was decided to deliver the TMS pulse 150 ms after the provision of visual feedback. Given the poor correlation between motor and phosphene thresholds in healthy individuals (Stewart et al., 2001; Boroojerdi et al., 2002), basing stimulation intensity on a percentage of the motor threshold may be inappropriate when stimulating nonmotor areas. As such, a number of TMS studies targeting the parietal cortex have used a fixed stimulation intensity corresponding to 60% of stimulator output (Dambeck et al., 2006; Vesia et al., 2006, 2008; Prime et al., 2008). In the present study, stimulation intensity was set at 70% of stimulator output.

### Experimental design and statistical analyses

Eight dependent variables (RT, MT, reach angle at peak velocity, reach angle variability at peak velocity, Final^x^, Final^x^ variability, Final^y^, and Final^y^ variability) were analyzed in the present study. For all analyses, permutation-based statistics were used, as this approach makes no assumptions about the underlying distribution of the data (Good, 2005; Maris and Oostenveld, 2007; Cohen, 2014). First, each variable was submitted to a three-way repeated-measures ANOVA with Group (between subject, two levels: P3 and P4), Target (within subject, two levels: right and left target), and Condition (within subject, five levels: NULL, R, RS, R + 1, and RS + 1) as factors. After obtaining the *F* ratios for the nonpermuted (i.e., true) data, the Group, Target, and Condition labels were randomly shuffled (in that order) across datasets, and permuted *F* ratios were obtained by applying a three-way repeated-measures ANOVA to the permuted datasets. This procedure (i.e., random permutation + three-way repeated-measures ANOVA) was repeated 5000 times to obtain a distribution of permuted *F* ratios, the majority of which should fall under the null hypothesis. Then, for each factor/interaction, a *p* value was obtained by way of a Monte-Carlo estimate. Briefly, this entailed dividing the number of permuted *F* ratios larger or equal to the *F* ratio obtained from the nonpermuted data by the total number of permutations (i.e., 5000). It should be noted here that, although the *p* values differed, the above-mentioned permutation analyses always led to the same statistical outcome when compared with a Greenhouse–Geisser adjusted parametric three-way repeated-measures ANOVA.

When a significant effect was found for any factor/interaction, *t* tests were used to identify differences between factor levels. When comparing between independent samples (i.e., group comparisons), independent *t* tests were used, whereas when comparing between dependent samples (i.e., Target or Condition comparisons), dependent *t* tests were used. Akin to the above-mentioned analyses, *t* test *p* values were obtained through permutation testing. Briefly, after obtaining *t* values from the nonpermuted datasets, a distribution of permuted *t* values was obtained for each contrast by randomly shuffling the factor labels and applying a *t* test to the permuted data for 5000 iterations. Thereafter, the number of permuted *t* values with an unsigned magnitude greater or equal to that of the true, nonpermuted *t* value (i.e., the values equally or more extreme) was divided by the total number of permutations to obtain a two-tailed *p* value. For all analyses, the threshold for significance was set at α = 0.05. When the breakdown of a significant factor/interaction required multiple paired comparisons, *p* values were Bonferroni corrected by multiplying them by the number of comparisons required to examine the significant factor/interaction. All Bonferroni corrections are specified in the Results section. Of note, despite yielding different *p* values, our *t* test permutation analyses always resulted in the same statistical outcome when compared with parametric *t* tests, even after Bonferroni correction.

To make the Results section more intuitive, if either left or right parietal stimulation impacts the postrotation bias, we would expect the direction-sensitive variables, notably reach angle at peak velocity, to show a Group × Target × Condition interaction, which would be driven by R + 1 versus RS + 1 differences at each target location. If both left and right parietal stimulation similarly impact the postrotation bias, we would expect the direction-sensitive variables to show a Target × Condition interaction, which would likewise be driven by R + 1 versus RS + 1 differences at each target location.

For all ANOVAs, effect sizes are reported as partial eta squared (η^2^*_p_*; Fritz et al., 2012; Lakens, 2013). Although clear η^2^*_p_* benchmarks are lacking for repeated-measures designs (Lakens, 2013), here we use the η^2^ benchmarks proposed by Cohen (1988) as an approximation for effect size amplitude. Specifically, η^2^*_p_* values > 0.01, 0.06, and 0.14 were respectively considered small, medium, and large. For all targeted comparisons (i.e., *post hoc t* tests), effect sizes were also calculated (independent samples, Cohen’s *d*; dependent samples, Cohen’s *dz*). Cohen’s *d* and *dz* values > 0.2, 0.5, and 0.8 are, respectively, considered small, medium, and large (Cohen, 1988; Field, 2009).

## Results

### Reach angle at peak velocity

**Figure 2.**
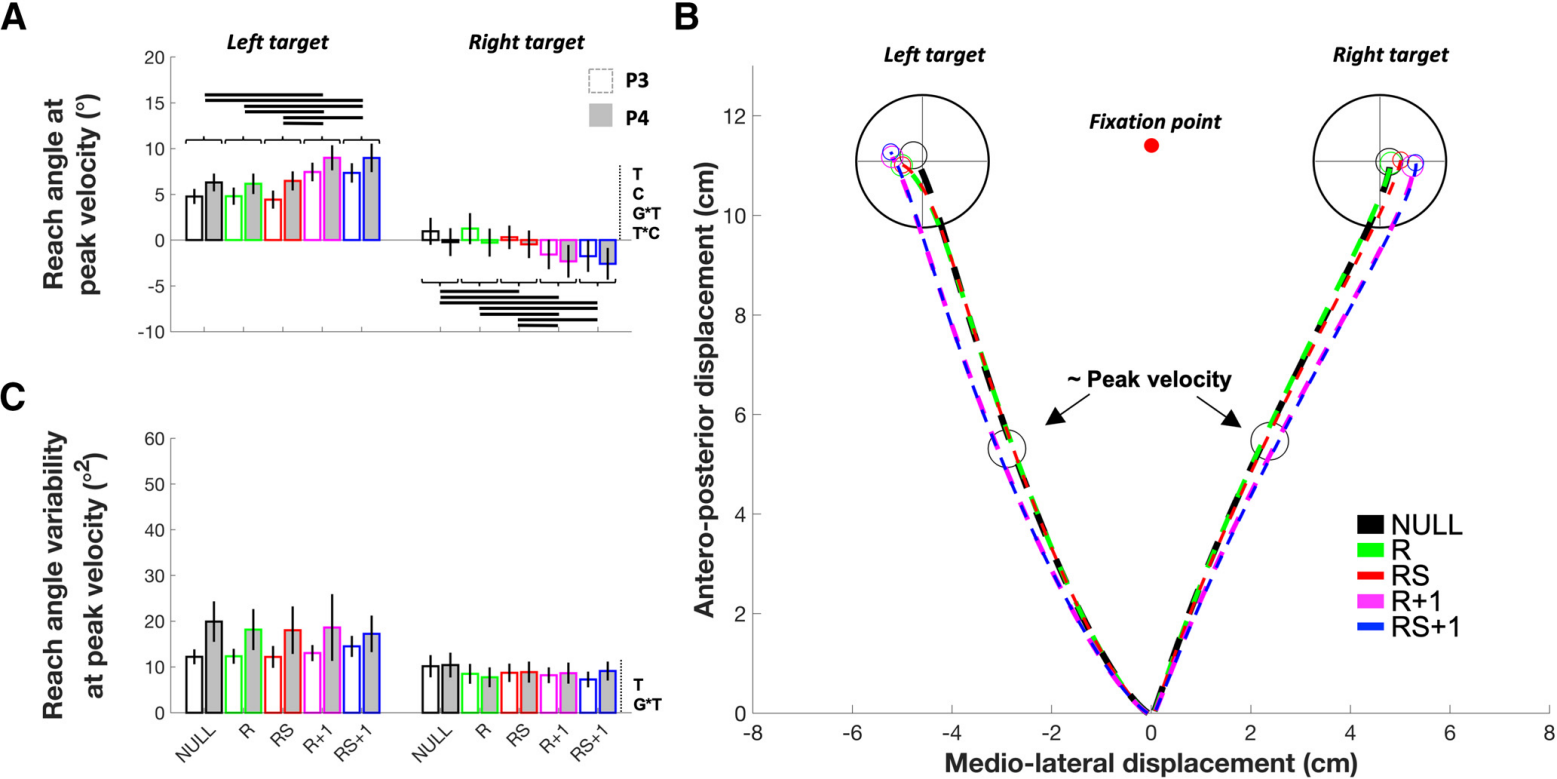
Hand direction data. ***A***, Reach angle at peak velocity. ***B***, Hand trajectories pooled across P3 and P4. ***C***, Reach angle variability at peak velocity. In ***A*** and ***C***, significant (*p *≤* *0.05) main effects and interactions are indicated to the right of the abscissa, where G, C, and T are abbreviations for Group, Condition, and Target, respectively. Horizontal links indicate significant differences (*p *≤* *0.05, Bonferroni corrected). Data are presented as the mean ± SEM.

For reach angle at peak velocity, significant effects were identified for Target (*F*_(1,26)_ = 148.77, *p *=* *0.000, η^2^*_p_* = 0.85), Condition (*F*_(4,104)_ = 2.76, *p *=* *0.031, η^2^*_p_* = 0.10), Target × Condition (*F*_(4,104)_ = 114.37, *p *=* *0.000, η^2^*_p_* = 0.81), and Group × Target (*F*_(1,26)_ = 4.94, *p *=* *0.035, η^2^*_p_* = 0.16), but not for Group (*F*_(1,26)_ = 0.17, *p *=* *0.676, η^2^*_p_* = 0.01), Group × Condition (*F*_(4,104)_ = 1.92, *p *=0.124, η^2^*_p_* = 0.07), or Group × Target × Condition (*F*_(4,104)_ = 0.23, *p *=* *0.922, η^2^*_p_* = 0.01; Fig. 2*A*). The Target main effect revealed that movements directed toward the left target showed a significant counterclockwise (i.e., positive) bias compared with movements directed toward the right target (Δ = 7.23°, *t*_(27)_ = 11.40, *p *=* *0.000, *dz *=* *2.15). This difference was likely a result of the dissimilar biomechanical requirements between left and right target reaches. Although there was a main effect of Condition, conditions were compared separately at each target location given the Target × Condition interaction. For left target trials, paired comparisons (Bonferroni correction: *p* × 10) showed a bias in hand direction following exposure to the visuomotor rotation, as reach angle at peak velocity in R + 1 and RS + 1 was shifted significantly more counterclockwise compared with NULL, R, and RS (Δ ≥ 2.63°, *t*_(27)_ ≥ 9.04, *p *≤* *0.002, *dz *≥* *1.71). In contrast to our hypothesis, however, there was no significant difference between R + 1 and RS + 1 trials (Δ = −0.05°, *t*_(27)_ = −0.23, *p *=* *1.00, *dz *=* *0.04). Moreover, no other contrast revealed a significant difference (Δ ≤ 0.09°, *t*_(27)_ ≤ 0.55, *p *=* *1.00, *dz *≤* *0.10). It should be noted here that for left target trials, 25 participants showed a counterclockwise bias in both R + 1 and RS + 1 compared with either NULL, R, and RS.

For right target trials, paired comparisons (Bonferroni correction: *p* × 10) also showed a bias in hand direction following exposure to the visuomotor rotation, as reach angle at peak velocity in R + 1 and RS + 1 was significantly more clockwise compared with NULL, R, and RS (Δ ≤ −1.87°, *t*_(27)_ ≤ −7.26, *p *≤* *0.002, *dz *≥* *1.37). In contrast to our hypothesis, however, there was no significant difference between R + 1 and RS + 1 trials (Δ = 0.23°, *t*_(27)_ = 1.31, *p *=* *1.00, *dz *=* *0.25). Additionally, a significant but marginal clockwise bias was found between RS and NULL (Δ = −0.44°, *t*_(27)_ = −3.27, *p *=* *0.30, *dz *=* *0.62). No other significant difference was found (Δ = −0.56° and 0.12°, *t*_(27)_ = −2.87 and 1.01, *p *≥* *0.097, *dz *=* *0.54). Of note, 25 participants showed a clockwise bias in both R + 1 and RS + 1 compared with either NULL, R, and RS, thus mirroring that observed for left target trials (see previous paragraph). To help illustrate the counterclockwise (left target) and clockwise (right target) hand biases observed in both R + 1 and RS + 1 trials, the mean movement trajectories across all participants are presented in Figure 2*B*. Overall, these results demonstrate that following a single rotation trial, a significant directional bias can be observed in the direction opposite to the rotation despite participants knowing that postrotation trials would not be perturbed.

Breakdown of the Group × Target interaction (Bonferroni correction: *p* × 2) revealed that for left target trials, the P4 group tended to show a greater counterclockwise bias compared with the P3 group (Δ = 1.62°, *t*_(26)_ = 2.21, *p *=* *0.072, *d *=* *0.83), although no intergroup differences were identified for right target trials (Δ = −1.01°, *t*_(26)_ = −0.90, *p *=* *0.75, *d *=* *0.34).

### Reach angle variability at peak velocity

For reach angle variability at peak velocity, significant effects were identified for Target (*F*_(1,26)_ = 33.56, *p *=* *0.000, η^2^*_p_* = 0.56) and Group × Target (*F*_(1,26)_ = 4.73, *p *=* *0.037, η^2^*_p_* = 0.15), but not for Group (*F*_(1,26)_ = 3.91, *p *=* *0.071, η^2^*_p_* = 0.13), Condition (*F*_(4,104)_ = 1.41, *p *=* *0.240, η^2^*_p_* = 0.05), Group × Condition (*F*_(4,104)_ = 0.43, *p *=* *0.79, η^2^*_p_* = 0.02), Target × Condition (*F*_(4,104)_ = 0.57, *p *=* *0.686, η^2^*_p_* = 0.02), or Group × Target × Condition (*F*_(4,104)_ = 1.44, *p *=* *0.232, η^2^*_p_* = 0.05; Fig. 2*C*). The Target effect indicated that reach angle at peak velocity was more variable for trials directed toward the left, compared with the right target (Δ = 6.86°^2^, *t*_(27)_ = 5.43, *p *=* *0.000, *dz *=* *1.03). Breakdown of the Group × Target interaction (Bonferroni correction: *p* × 2) revealed that reach angle at peak velocity was not significantly different between groups for reaches made toward the right target (Δ = 0.39°^2^, *t*_(26)_ = 0.32, *p *=* *1.00, *d *=* *0.12), but tended to be greater in P4 than in P3 for reaches made toward the left target (Δ = 5.54°^2^, *t*_(26)_ = 2.30, *p *=* *0.058, *d *=* *0.87).

### Final^x^

**Figure 3.**
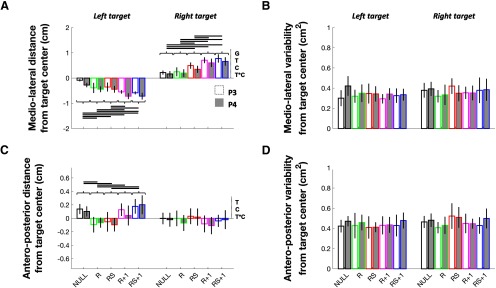
Final hand position data. ***A***, Final^x^. ***B***, Final^x^ variability. ***C***, Final anteroposterior hand position (Final^y^). ***D***, Final^y^ variability. Significant (*p *≤* *0.05) main effects and interactions are indicated to the right of the abscissa, where G, C, and T are abbreviations for Group, Condition, and Target, respectively. Horizontal links indicate a significant difference (*p *≤* *0.05, Bonferroni corrected). Data are presented as the mean ± SEM.

For Final^x^, significant effects were identified for Group (*F*_(1,26)_ = 5.38, *p *=* *0.039, η^2^*_p_* = 0.17), Target (*F*_(1,26)_ = 244.62, *p *=* *0.000, η^2^*_p_* = 0.90), Condition (*F*_(4,104)_ = 7.19, *p *=* *0.000, η^2^*_p_* = 0.22), and Target × Condition (*F*_(4,104)_ = 58.97, *p *=* *0.000, η^2^*_p_* = 0.69), but not for Group × Target (*F*_(1,26)_ = 0.08, *p *=* *0.772, η^2^*_p_* = 0.00), Group × Condition (*F*_(4,104)_ = 0.78, *p *=* *0.534, η^2^*_p_* = 0.03), or Group × Target × Condition (*F*_(4,104)_ = 0.44, *p *=* *0.781, η^2^*_p_* = 0.02; Fig. 3*A*). The Group effect revealed that Final^x^ was generally biased to the left of either target in P4 compared with P3 (Δ = −0.11 cm, *t*_(26)_ = −2.32, *p *=* *0.028, *d *=* *0.87), whereas the Target effect indicated that, overall, participants terminated their movements at the left of the target on left target trials and at the right of the target on right target trials (Δ = −0.90 cm, *t*_(27)_ = −15.91, *p *=* *0.000, *dz *=* *3.01). Although the Group effect was arguably the result of an inherent group sampling difference, the Target effect was likely due to the different biomechanical requirements for left and right target reaches.

Although there was a main effect of Condition, conditions were compared separately at each target location given the Target × Condition interaction. In line with the reach angle at peak velocity results, left target paired comparisons (Bonferroni correction: *p* × 10) revealed a directional bias following exposure to the visuomotor rotation, with R + 1 and RS + 1 being significantly leftward compared with all other conditions (Δ ≤ −0.22 cm, *t*_(27)_ ≤ −3.40, *p *≤* *0.046, *dz *≥* *0.64). Additionally, R and RS showed a significant leftward bias when compared with NULL (Δ ≤ −0.22 cm, *t*_(27)_ ≤ −4.05, *p *≤* *0.01, *dz *≥* *0.77). No significant Final^x^ differences were found between R and RS (Δ = −0.02 cm, *t*_(27)_ = −0.49, *p *=* *1.00, *dz *=* *0.09) or between R + 1 and RS + 1 (Δ = 0.02 cm, *t*_(27)_ = 0.48, *p *=* *1.00, *dz *=* *0.09). For right target trials, paired comparisons (Bonferroni correction: *p* × 10) also revealed a directional bias, with R + 1 and RS + 1 Final^x^ being significantly rightward compared with all other conditions (Δ ≥ 0.23 cm, *t*_(27)_ ≥ 5.28, *p *≤* *0.002, *dz *≥* *1.00). Moreover, right target RS trials showed a significant rightward bias compared with both NULL and R trials (Δ ≥ 0.20 cm, *t*_(27)_ ≥ 5.00, *p *≤* *0.002, *dz *≥* *0.94). No other contrast revealed a significant difference (Δ = −0.06 and −0.03] cm, *t*_(27)_ = −1.59 and −0.87, *p *=* *1.00, *dz *≤* *0.30). In sum, Final^x^ differences in R + 1 and RS + 1 compared with all other conditions at both target locations corroborate the results obtained for reach angle at peak velocity. In contrast, the leftward biases observed on left target R and RS trials compared with NULL may have resulted from the initiation of an online correction following perturbation detection. The curvature near the end of the left target R and RS trajectories (Fig. 2*A*, left target), coupled with the lack of statistically significant reach angle at peak velocity differences between left target NULL, R, and RS trials, would support such an interpretation. Interestingly, although a significant rightward right target Final^x^ bias was found in RS compared with NULL, evidence of online correction was less evident on right compared with left target trials (Fig. 2*A*, right target). This may be explained by the fact that MTs were significantly shorter for right than left target reaches (see MT), which may have limited the ability of participants to implement an online correction on right target R and RS trials.

### Final^x^ variability

No significant effects were found for Final^x^ variability (Group: *F*_(1,26)_ = 0.20, *p *=* *0.653, η^2^*_p_* = 0.01; Target: *F*_(1,26)_ = 1.08, *p *=* *0.314, η^2^*_p_* = 0.04; Condition: *F*_(4,104)_ = 1.71, *p *=* *0.148, η^2^*_p_* = 0.06; Group × Target: *F*_(1,26)_ = 0.77, *p *=* *0.377, η^2^*_p_* = 0.03; Group × Condition: *F*_(4,104)_ = 1.76, *p *=* *0.139, η^2^*_p_* = 0.06; Target × Condition: *F*_(4,104)_ = 0.65, *p *=* *0.622, η^2^*_p_* = 0.02; Group × Target × Condition: *F*_(4,104)_ = 0.55, *p *=* *0.708, η^2^*_p_* = 0.02; Fig. 3*B*).

### Final^y^

For Final^y^, significant effects were identified for Target (*F*_(1,26)_ = 8.86, *p *=* *0.008, η^2^*_p_* = 0.25), Condition (*F*_(4,104)_ = 6.76, *p *=* *0.000, η^2^*_p_* = 0.21), and Target × Condition (*F*_(4,104)_ = 8.70, *p *=* *0.000, η^2^*_p_* = 0.25), but not for Group (*F*_(1,26)_ = 0.18, *p *=* *0.657, η^2^*_p_* = 0.01), Group × Target (*F*_(1,26)_ = 0.00, *p *=* *0.991, η^2^*_p_* = 0.00), Group × Condition (*F*_(4,104)_ = 0.40, *p *=* *0.810, η^2^*_p_* = 0.02), or Group × Target × Condition (*F*_(4,104)_ = 0.40, *p *=* *0.809, η^2^*_p_* = 0.02; Fig. 3*C*). The Target effect indicated that hand displacement along the *y*-axis was generally farther on left, compared with right target trials (Δ = 0.08 cm, *t*_(27)_ = 3.03, *p *=* *0.005, *dz *=* *0.57). Although there was a main effect of Condition, conditions were compared separately at each target location given the Target × Condition interaction. For the left target, although paired comparisons (Bonferroni correction: *p* × 10) did not reveal a significant difference between R and RS (Δ = −0.006 cm, *t*_(27)_ = −0.16, *p *=* *1.00, *dz *=* *0.03), Final^y^ was significantly closer to the start base in these two conditions compared with NULL and RS + 1 (Δ ≤ −0.19 cm, *t*_(27)_ ≤ −4.44, *p *≤* *0.002, *dz *≥* *0.84). Final^y^ was also significantly farther from the start base in R + 1 compared with R (Δ = 0.16 cm, *t*_(27)_ = 3.07, *p *=* *0.050, *dz *=* *0.58), but not compared with RS (Δ = 0.16 cm, *t*_(27)_ = 2.79, *p *=* *0.074, *dz *=* *0.53). No significant differences were observed between NULL, R + 1 and RS + 1 (Δ = −0.11 and 0.04 cm, *t*_(27)_ = −2.74 and 0.93, *p *≥* *0.110, *dz *≤* *0.52). Of note, and in contrast to left target trials, no intercondition Final^y^ differences (Bonferroni correction: *p* × 10) were observed for right target trials (Δ = −0.07 and 0.12 cm, *t*_(27)_ = −1.73 and 2.20, *p *≥* *0.382, *dz *≤* *0.42). Of note, the fact that significant (or close to significant) Final^y^ differences were only observed between left target rotation and nonrotation trials supports the idea that some form of online correction was implemented during left target rotation trials.

### Final^y^ variability

No significant effects were found for Final^y^ variability (Group: *F*_(1,26)_ = 0.35, *p *=* *0.550, η^2^*_p_* = 0.01; Target: *F*_(1,26)_ = 1.41, *p *=* *0.245, η^2^*_p_* = 0.05; Condition: *F*_(4,104)_ = 0.68, *p *=* *0.604, η^2^*_p_* = 0.03; Group × Target: *F*_(1,26)_ = 0.03, *p *=* *0.856, η^2^*_p_* = 0.00; Group × Condition: *F*_(4,104)_ = 0.58, *p *=* *0.688, η^2^*_p_* = 0.02; Target × Condition: *F*_(4,104)_ = 2.03, *p *=* *0.093, η^2^*_p_* = 0.07; Group × Target × Condition: *F*_(4,104)_ = 0.08, *p *=* *0.990, η^2^*_p_* = 0.00; Fig. 3*D*).

### RT

**Figure 4.**
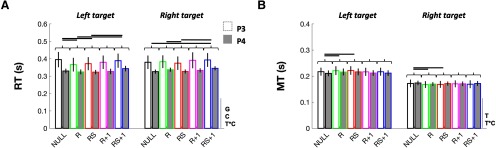
Movement onset and duration data. ***A***, RT. ***B***, MT. Significant (*p *≤* *0.05) main effects and interactions are indicated to the right of the abscissa, where G, C, and T are abbreviations for Group, Condition, and Target, respectively. Horizontal links indicate a significant difference (*p *≤* *0.05, Bonferroni corrected). Data are presented as the mean ± SEM.

For RT, significant effects were identified for Group (*F*_(1,26)_ = 6.10, *p *=* *0.026, η^2^*_p_* = 0.19), Condition (*F*_(4,104)_ = 10.36, *p *=* *0.000, η^2^*_p_* = 0.28), and Target × Condition (*F*_(4,104)_ = 5.79, *p *=* *0.000, η^2^*_p_* = 0.18), but not for Target (*F*_(1,26)_ = 1.22, *p *=* *0.283, η^2^*_p_* = 0.04), Group × Target (*F*_(1,26)_ = 0.00, *p *=* *0.989, η^2^*_p_* = 0.00), Group × Condition (*F*_(4,104)_ = 2.32, *p *=* *0.061, η^2^*_p_* = 0.08), or Group × Target × Condition (*F*_(4,104)_ = 0.85, *p *=* *0.510, η^2^*_p_* = 0.03; Fig. 4*A*). The Group effect revealed that RTs were significantly shorter in P4 compared with P3 (Δ = −0.051 s, *t*_(26)_ = −2.47, *p *=* *0.020, *d *=* *0.93). Although there was a main effect of Condition, conditions were compared separately at each target location given the Target × Condition interaction. For the left target, paired comparisons (Bonferroni correction: *p* × 10) revealed that RTs in NULL and RS + 1 were significantly longer compared with both R and RS (Δ ≥ 0.015 s, *t*_(27)_ ≥ 2.79, *p *≤* *0.002, *dz *≥* *0.53). No other paired comparison revealed a significant difference for left target trials (Δ = −0.013 and 0.009 s, *t*_(27)_ = −2.91 and 1.72, *p *≥* *0.086, *dz *≤* *0.55). For right target trials, paired comparisons (Bonferroni correction: *p* × 10) revealed that RTs in RS + 1 were significantly longer compared with NULL and RS (Δ ≥ 0.16 s, *t*_(27)_ ≥ 5.31, *p *≤* *0.002, *dz *≥* *1.00). Additionally, RTs were significantly, but marginally, longer in R compared with RS (Δ = 0.009 s, *t*_(27)_ = −4.02, *p *=* *0.010, *dz *=* *0.76). No other contrast yielded statistically significant RT differences for right target trials (Δ = −0.011 and 0.002 s, *t*_(27)_ = −2.56 and 0.77, *p *≥* *0.11, *dz *≤* *0.48). Hence, overall, participants were somewhat slower to respond on RS + 1 trials, perhaps because they were distracted by the tactile sensation and “clicking” sound made by the stimulator on previous RS trials.

### MT

For MT, significant effects were identified for Target (*F*_(1,26)_ = 321.83, *p *=* *0.000, η^2^*_p_* = 0.93) and Target × Condition (*F*_(4,104)_ = 7.32, *p *=* *0.000, η^2^*_p_* = 0.22), but not for Group (*F*_(1,26)_ = 0.04, *p *=* *0.842, η^2^*_p_* = 0.00), Condition (*F*_(4,104)_ = 2.07, *p *=* *0.095, η^2^*_p_* = 0.07), Group × Target (*F*_(1,26)_ = 2.04, *p *=* *0.163, η^2^*_p_* = 0.07), Group × Condition (*F*_(4,104)_ = 0.22, *p *=* *0.932, η^2^*_p_* = 0.01), or Group × Target × Condition (*F*_(4,104)_ = 0.27, *p *=* *0.904, η^2^*_p_* = 0.01; Fig. 4*B*). The Target effect indicated that MTs were significantly shorter on right than left target trials (Δ = −0.046 s, *t*_(27)_ = −17.60, *p *=* *0.000, *dz *=* *3.33). To break down the Target × Condition interaction, we compared all conditions separately at each target location. For left target trials, paired comparisons (Bonferroni correction: *p* × 10) revealed that R and RS MTs were significantly, though marginally, longer compared with NULL trials (Δ ≥ 0.0047 s, *t*_(27)_ ≥ 4.01, *p *≤* *0.006, *dz *≥* *0.76), with no other contrast yielding a significant difference (Δ = −0.00047 and 0.0047 s, *t*_(27)_ = −0.76 and 2.84, *p *≥* *0.074, *dz *≤* *0.54). In contrast, for right target trials, paired comparisons (Bonferroni correction: *p* × 10) revealed that MTs were significantly, though marginally, longer in NULL compared with R and RS (Δ ≥ 0.003 s, *t*_(27)_ ≥ 3.32, *p *≤* *0.014, *dz *≥* *0.63), with no other contrast reaching the significance threshold (Δ = −0.0019 and 0.003 s, *t*_(27)_ = −1.43 and 2.10, *p *≥* *0.42, *dz *≤* *0.40).

## Discussion

A number of studies have shown that exposure to intermittent or randomly changing sensorimotor perturbations bias subsequent motor behavior (Diedrichsen et al., 2005; Galea et al., 2015; Torrecillos et al., 2015; Tan et al., 2016; Savoie et al., 2018). In a recent EEG study, Savoie et al. (2018) observed increased activity over parietal regions 140–260 ms after the provision of rotated visual feedback, raising the possibility that the parietal cortex is involved in the error processing that leads to the emergence of this bias. The goal of the present study was to investigate this. To do so, participants made rapid reaching movements while being pseudorandomly exposed to a visuomotor rotation with, or without, TMS stimulation over the parietal cortex 150 ms after movement onset. The reasoning was that if the parietal responses observed by Savoie et al. (2018) were causally involved in the subsequent change in motor output, TMS stimulation would reduce the postrotation bias. Although a potent directional reaching bias was indeed observed following rotated trials in the present study, the TMS protocol did not meaningfully impact it. Therefore, the present results suggest that ∼150 ms after initiating a closed-loop movement under visuomotor rotation, the activity of the parietal areas located under P3 and P4 may not be critical for the emergence of visuomotor rotation-induced changes in motor output.

In the present study, we refer to the change in movement direction that follows single-trial exposure to a visuomotor rotation as a postrotation bias. Technically, this bias differs from classic sensorimotor adaptation in that the latter typically involves performance improvements that accumulate over several trials. Despite this difference, it seems likely that the mechanisms that subtend both the postrotation bias reported in this study and classic adaptation are related (Donchin et al., 2003). In the present study, we further speculate that the postrotation directional bias was driven by mechanisms related to implicit adaptation, as participants were asked to aim straight for the cued target regardless of what the cursor did. Obviously, given that we did not ask participants to report where they were aiming prior to each trial (Taylor et al., 2014), we cannot ascertain that participants complied with these instructions and, thus, cannot guarantee that no strategy was used. It should be noted, however, that the (absolute) bias measured in R + 1 and RS + 1 trials ranged from 2.31° to 2.68° (compared with NULL). Given that the errors experienced on R and RS trials were approximately the same size as the rotation itself (i.e., 45°), participants compensated for ∼5% of the error experienced on these rotated trials. The amplitude of these perturbation-induced changes in reach direction are consistent with the learning rate that should be expected for implicit visuomotor adaptation to a 45° rotation, which has been reported to hover between 2% and 10% (McDougle et al., 2015). Hence, although we cannot confirm that the observed postrotation bias was driven by implicit adaptation mechanisms, it appears a likely possibility.

According to anatomic labeling studies, P3 and P4, respectively, lie over left and right posterior areas of the inferior parietal lobule, presumably corresponding to the angular gyrus and adjacent part of the intraparietal sulcus (Homan et al., 1987; Herwig et al., 2003; Okamoto et al., 2004; Vesia et al., 2008). These regions are tightly involved in motor planning and control, as TMS over P3 and P4 during reach planning has been shown to bias reach kinematics (Vesia et al., 2006, 2008, 2010) and to increase ipsilateral motor cortex excitability at rest (Koch et al., 2007) and during reach planning toward contralateral visual targets (Koch et al., 2008). Concerning sensorimotor adaptation, activity in these areas has been shown to be modulated in response to visuomotor perturbations (Graydon et al., 2005; Girgenrath et al., 2008; Luauté et al., 2009; Chapman et al., 2010) suggesting that they arguably play a role in this type of learning. Despite this, TMS stimulation over P3 or P4 had no effect on the postrotation bias. Assuming that the bias observed in R + 1 and RS + 1 trials can be used as a single-trial proxy for implicit adaptation, the present data suggest that the inferior parietal areas targeted by the TMS protocol are not involved in implicit adaptation 150 ms after movement onset. This begs the question: what roles do the parietal areas located under P3 and P4 play during visuomotor error processing? In the following paragraphs, we explore three possibilities, starting from the most to the least plausible based on the amount of supporting evidence.

### What might parietal responses to a visuomotor rotation mean?

The timing of the parietal responses to visuomotor errors reported by Savoie et al. (2018; starting at ∼140 ms) is consistent with the putative time required for macaque parietal neurons to encode new movement kinematics following a target jump (∼150 ms, Archambault et al., 2009). Thus, a first possibility is that early parietal activations during visuomotor adaptation relate to the engagement of automatic online correction mechanisms (Pisella et al., 2000). Indeed, there is compelling evidence showing that the parietal cortex is a crucial brain area for online corrections during reaching (Desmurget et al., 1999; Pisella et al., 2000; Buiatti et al., 2013), which can be observed when individuals reach, rather than shoot through, visual targets under visuomotor rotation (Diedrichsen et al., 2005; Seidler et al., 2006; Seidler and Noll, 2008; Torrecillos et al., 2015). Given that it is thought to continuously compare the position of the hand and target during reaching (Buneo et al., 2002; Diedrichsen et al., 2005; Buneo and Andersen, 2006), the parietal cortex may play an important role in the detection of performance errors, which both engage online correction mechanisms and drive explicit adaptation (Taylor and Ivry, 2011; Taylor et al., 2014). Interestingly, it has been proposed that corrective motor responses could serve as teaching signals for sensorimotor adaptation (Albert and Shadmehr, 2016; Shadmehr, 2018). For instance, Della-Maggiore et al. (2004) have shown that precluding corrective movements by interfering (single-pulse TMS) with the medial bank of the left intraparietal sulcus impairs performance during force-field adaptation. However, the authors reported little to no impact of parietal stimulation on short term retention (i.e., aftereffects), which is thought to reflect implicit adaptation (Mazzoni and Krakauer, 2006; Taylor et al., 2014; Morehead et al., 2017). Hence, although the parietal cortex may not play a direct role in implicit adaptation, the corrective movements it mediates may serve as learning templates for explicit adaptation.

Another possible reason why the stimulation of P3 or P4 did not impact the postrotation bias is that the targeted parietal areas may be involved in the storage of motor memories rather than immediate performance improvements, akin to the presumed role of the primary motor cortex in adaptation (Richardson et al., 2006; Hadipour-Niktarash et al., 2007; Overduin et al., 2009; Galea et al., 2011; Hamel et al., 2017). Indeed, Galea et al. (2011) showed that increasing excitability of the primary motor cortex prior to visuomotor adaptation had no bearing on performance during acquisition, but improved short-term retention. Likewise, Hamel et al. (2017) showed that disrupting the primary motor cortex with single-pulse TMS during visuomotor adaptation did not impact performance during acquisition, but reduced retention 24 h later. Although data suggesting that the same applies for the parietal cortex are scarce, it is plausible that parietal areas might subserve adaptation in a similar way. For instance, computational modeling work by Tanaka et al. (2009) suggests that visuomotor remapping occurs by way of synaptic reweighting between posterior parietal and motor areas, raising the possibility that, together, these areas could form a network that subtends retention. Empirical evidence for this has recently been provided by Della-Maggiore et al. (2017), who observed increased functional connectivity within a motor network comprising the supramarginal gyrus ∼6 h after visuomotor adaptation. The fact that the strength of this network correlated with long-term retention led the authors to suggest that it reflected the stabilization of motor memories following adaptation. More direct evidence for inferior parietal involvement in visuomotor memory retention comes from a study by Moisello et al. (2015), who demonstrated that applying excitatory (5 Hz) repetitive TMS over electrode P6 after visuomotor adaptation robustly improved 24 h retention in Parkinson’s patients. Thus, a reasonable proposition is that the parietal cortex mediates the early formation of a visuomotor memory rather than trial-per-trial changes in motor output, which might explain why TMS stimulation had no impact on the postrotation directional reaching bias.

Finally, another possibility is that inferior parietal areas may contribute to proprioceptive realignment, which is known to occur during visuomotor adaptation (Simani et al., 2007; Cressman and Henriques, 2009; Izawa and Shadmehr, 2011; Salomonczyk et al., 2011, 2012; Nikooyan and Ahmed, 2015), rather than to immediate adjustments in motor behavior. This possibility was first raised by Clower et al. (1996), who reported concomitant proprioceptive recalibration and increased cerebral blood flow along the lateral bank of the intraparietal sulcus (i.e., supramarginal/angular gyrus) when participants made reaching movements while wearing displacing prisms. More recently, Block et al. (2013) provided direct support for this by showing that the application of inhibitory theta burst stimulation over the angular gyrus disrupts the relationship between visuo-proprioceptive weighting and realignment to shifted visual feedback. Interestingly, Cressman and Henriques (2010) have shown that proprioceptive realignment can occur in response to passively presented visuo-proprioceptive incongruities (Cressman and Henriques, 2010). In this light, perhaps the parietal cortex responds to cross-sensory errors between vision and proprioception (Henriques and Cressman, 2012), rather than efference copy-based SPEs. This should be tested in future studies.

### Limitations

It should be remembered that the characteristics of the stimulation protocol used herein constrain the inferential power of the results. First, despite the null impact of P3 or P4 stimulation on the postrotation bias, the present results cannot rule out the potential involvement of more rostral or medial parietal nodes in this phenomenon. For instance, neuroimaging work has also shown that visuomotor perturbations increase activity within the superior parietal lobule (Inoue et al., 1997, 2000; Ghilardi et al., 2000; Krakauer et al., 2004; Diedrichsen et al., 2005; Graydon et al., 2005; Luauté et al., 2009; Chapman et al., 2010), making areas 5 and 7 possible neural substrates for motor output changes following exposure to a perturbation. Further evidence for this comes from a recent study in monkeys showing that microstimulation of area 5 neurons in the vicinity of the intraparietal sulcus results in iterative, adaptation-like changes in reach behavior (Inoue and Kitazawa, 2018). Second, the interpretation of the present results is limited to the purported duration of the TMS effect. Given that cortical activity is thought to be disrupted for a few hundred milliseconds following single-pulse TMS (Premoli et al., 2014; Chung et al., 2015), stimulating 150 ms after movement onset arguably covered the putative time window of SPE processing (Savoie et al., 2018). However, it must be remembered that the timing of the differences reported by Savoie et al. (2018) were based on the average of all participants. Thus, it is possible that the TMS pulse was delivered too early or late in some participants of the present study. Stimulation timing issues could be addressed in future studies by lengthening the stimulation period using online repetitive TMS (Vesia et al., 2010), which could be set to cover a wider time window for parietal involvement in error processing (e.g., 0–500 ms).

### Conclusion

Although the present results must be interpreted in light of the spatiotemporal characteristics of the TMS protocol used, they suggest that the parietal regions lying under P3 and P4 do not causally contribute to the directional reaching bias that takes place following single-trial exposure to a visuomotor rotation. A number of questions remain concerning the roles of the parietal cortex in adaptive behavior. Among these are its potential involvement in online control, the formation of motor memories, and proprioceptive recalibration.

## Synthesis

Reviewing Editor: Trevor Drew, University of Montreal Decisions are customarily a result of the Reviewing Editor and the peer reviewers coming together and discussing their recommendations until a consensus is reached. When revisions are invited, a fact-based synthesis statement explaining their decision and outlining what is needed to prepare a revision will be listed below. The following reviewer(s) agreed to reveal their identity: Yves Rossetti.

Reviewer#1

It seems like authors have carefully considered all the comments made in the first step of the peer-reviewing process. The authors appropriately adjusted the rationale of their study, enriched their analysis of the literature and specified the aim of their work so as to remove any semantic ambiguity concerning the notion of implicit adaptation.

The authors also made efforts to clarify some methodological aspects and judiciously re-organized their discussion part.

To my mind, the only remaining concern refers to the instructions given to the subjects in order to avoid the use of any strategy to reach to target (i.e. to ignore the cursor trajectories). First, as mentioned by Reviewer 2, it is possible that not all the subjects followed these instructions. Additionally, even if they did follow these instructions, it is not guaranteed that no explicit component is contained in the post rotation bias despite authors’ assumption.

Nevertheless, it appears that the authors carefully specified these limitations and qualified some aspects of their methodology. Notably, they specified that despite their effort to favour the implication of implicit processes, they cannot assume that no explicit component was measured.

In that respect, I do not feel that these limitations represent a big concern to the extent that authors modified the very aim of their study. Thus, these limitations do not minimize the empirical and theoretical contributions of this work.

-

Reviewer#2

The manuscript provides negative findings presumably regarding a role of posterior parietal cortex in ‘implicit adaptation’ to visuomotor rotations. However, this does not provide conclusive negative results for the following reasons: 1) The paradigm tests a N-1, N+1, single perturbation effect, not adaptation. 2) The paradigm provides single pulse localized TMS 150 ms following visual feedback. This simply shows that this temporally specific and spatially specific input doesn’t affect the responses tested, not that PPC is not involved in adaptation. Previous lesion studies have implicated PPC in visual motor adaptation, including abolishing after-effects, which are thought to reflect implicit learning.

The authors have responded to my previous concerns. However, many aspects of these responses were not adequate:

1. The removal of 10% of the data due to ‘outlier’ status is inappropriate and artificially removes variance from the data. All trials should be included in analysis, especially given that only perturbation (+- 1) are included. This is an artificial manipulation of the data. All data should be included in the statistical analysis. Only trials in which the participants did not respond to the go signal should be removed. It is also not clear whether removal of 10% of trials was consistent across subjects, or whether the data of some subjects was essentially removed.

2. The paradigm does not convincingly address visual motor adaptation, as reflected by previous literature, but rather this paradigm addresses response to visual motor perturbations by assessing the post-perturbation ‘aftereffect’. No adaptation took place during this paradigm.

3. Previous literature has convincingly demonstrated that when PPC (intraparietal sulcus) is lesioned, both implicit and explicit adaptation is abolished (Mutha et al. - 2 studies). This leaves open the question of what this study is testing.

4. The negative results of the current study do not validate that posterior parietal cortex is NOT involved in visual motor adaptation for the following reasons:

a) The paradigm does not test visual motor adaptation but tests the aftereffects from a perturbation. The two concepts should not be confused. This is NOT an adaptation paradigm, but rather a single trial perturbation paradigm.

b) The lack of effects of single pulse TMS 150 ms following feedback does not indicate that PPC is not involved in this perturbation response. Instead it indicates that this specific location of PPC when stimulated at this particular time does not disrupt this perturbation response.

The generalization of the interpretation of results to visual motor adaptation and to a role of PPC in implicit adaptation is inappropriate, and does not reflect t

## References

[B1] Albert ST, Shadmehr R (2016) The neural feedback response to error as a teaching signal for the motor learning system. J Neurosci 36:4832–4845. 10.1523/JNEUROSCI.0159-16.2016 27122039 PMC4846676

[B2] Archambault PS, Caminiti R, Battaglia-Mayer A (2009) Cortical mechanisms for online control of hand movement trajectory: the role of the posterior parietal cortex. Cereb Cortex 19:2848–2864. 10.1093/cercor/bhp058 19359349

[B3] Block H, Bastian A, Celnik P (2013) Virtual lesion of angular gyrus disrupts the relationship between visuoproprioceptive weighting and realignment. J Cogn Neurosci 25:636–648. 10.1162/jocn_a_00340 23249345 PMC3750114

[B4] Boroojerdi B, Meister IG, Foltys H, Sparing R, Cohen LG, Töpper R (2002) Visual and motor cortex excitability: a transcranial magnetic stimulation study. Clin Neurophysiol 113:1501–1504. 10.1016/s1388-2457(02)00198-0 12169333

[B5] Brainard DH (1997) The psychophysics toolbox. Spat Vis 10:433–436. 10.1163/156856897X00357 9176952

[B6] Buiatti T, Skrap M, Shallice T (2013) Reaching a moveable visual target: dissociations in brain tumour patients. Brain Cogn 82:6–17. 10.1016/j.bandc.2013.02.004 23501699

[B7] Buneo CA, Jarvis MR, Batista AP, Andersen RA (2002) Direct visuomotor transformations for reaching. Nature 416:632–636. 10.1038/416632a 11948351

[B8] Buneo CA, Andersen RA (2006) The posterior parietal cortex: sensorimotor interface for the planning and online control of visually guided movements. Neuropsychologia 44:2594–2606. 10.1016/j.neuropsychologia.2005.10.011 16300804

[B9] Chapman HL, Eramudugolla R, Gavrilescu M, Strudwick MW, Loftus A, Cunnington R, Mattingley JB (2010) Neural mechanisms underlying spatial realignment during adaptation to optical wedge prisms. Neuropsychologia 48:2595–2601. 10.1016/j.neuropsychologia.2010.05.006 20457170

[B10] Chung SW, Rogasch NC, Hoy KE, Fitzgerald PB (2015) Measuring brain stimulation induced changes in cortical properties using TMS-EEG. Brain Stimul 8:1010–1020. 10.1016/j.brs.2015.07.029 26275346

[B11] Clower DM, Hoffman JM, Votaw JR, Faber TL, Woods RP, Alexander GE (1996) Role of posterior parietal cortex in the recalibration of visually guided reaching. Nature 383:618–621. 10.1038/383618a0 8857536

[B12] Cohen J (1988) Statistical power analysis for the behavioral sciences, Ed 2 Hillsdale, NJ: Erlbaum.

[B13] Cohen MX (2014) Analyzing neural time series data: theory and practice. Cambridge, MA: MIT.

[B14] Cressman EK, Henriques DY (2009) Sensory recalibration of hand position following visuomotor adaptation. J Neurophysiol 102:3505–3518. 10.1152/jn.00514.2009 19828727

[B15] Cressman EK, Henriques DY (2010) Reach adaptation and proprioceptive recalibration following exposure to misaligned sensory input. J Neurophysiol 103:1888–1895. 10.1152/jn.01002.2009 20130036

[B16] Dambeck N, Sparing R, Meister IG, Wienemann M, Weidemann J, Topper R, Boroojerdi B (2006) Interhemispheric imbalance during visuospatial attention investigated by unilateral and bilateral TMS over human parietal cortices. Brain Res 1072:194–199. 10.1016/j.brainres.2005.05.075 16426588

[B17] Della-Maggiore V, Malfait N, Ostry DJ, Paus T (2004) Stimulation of the posterior parietal cortex interferes with arm trajectory adjustments during the learning of new dynamics. J Neurosci 24:9971–9976. 10.1523/JNEUROSCI.2833-04.2004 15525782 PMC6730240

[B18] Della-Maggiore V, Villalta JI, Kovacevic N, McIntosh AR (2017) Functional evidence for memory stabilization in sensorimotor adaptation: a 24-h resting-state fMRI study. Cereb Cortex 27:1748–1757. 10.1093/cercor/bhv289 26656723

[B19] Desmurget M, Epstein CM, Turner RS, Prablanc C, Alexander GE, Grafton ST (1999) Role of the posterior parietal cortex in updating reaching movements to a visual target. Nat Neurosci 2:563–567. 10.1038/9219 10448222

[B20] Diedrichsen J, Hashambhoy Y, Rane T, Shadmehr R (2005) Neural correlates of reach errors. J Neurosci 25:9919–9931. 10.1523/JNEUROSCI.1874-05.2005 16251440 PMC1479774

[B21] Donchin O, Francis JT, Shadmehr R (2003) Quantifying generalization from trial-by-trial behavior of adaptive systems that learn with basis functions: theory and experiments in human motor control. J Neurosci 23:9032–9045. 14534237 10.1523/JNEUROSCI.23-27-09032.2003 PMC6740843

[B22] Field AP (2009) Discovering statistics using SPSS: (and sex, drugs and rock ‘n’ roll), Ed 3 Los Angeles: SAGE.

[B23] Fritz CO, Morris PE, Richler JJ (2012) Effect size estimates: current use, calculations, and interpretation. J Exp Psychol Gen 141:2–18. 10.1037/a0024338 21823805

[B24] Galea JM, Vazquez A, Pasricha N, de Xivry JJ, Celnik P (2011) Dissociating the roles of the cerebellum and motor cortex during adaptive learning: the motor cortex retains what the cerebellum learns. Cereb Cortex 21:1761–1770. 10.1093/cercor/bhq246 21139077 PMC3138512

[B25] Galea JM, Mallia E, Rothwell J, Diedrichsen J (2015) The dissociable effects of punishment and reward on motor learning. Nat Neurosci 18:597–602. 10.1038/nn.3956 25706473

[B26] Ghilardi M, Ghez C, Dhawan V, Moeller J, Mentis M, Nakamura T, Antonini A, Eidelberg D (2000) Patterns of regional brain activation associated with different forms of motor learning. Brain Res 871:127–145. 10.1016/s0006-8993(00)02365-9 10882792

[B27] Girgenrath M, Bock O, Seitz RJ (2008) An fMRI study of brain activation in a visual adaptation task: activation limited to sensory guidance. Exp Brain Res 184:561–569. 10.1007/s00221-007-1124-8 17909772

[B28] Good PI (2005) Permutation, parametric and bootstrap tests of hypotheses, Ed 3 New York: Springer.

[B29] Graydon FX, Friston KJ, Thomas CG, Brooks VB, Menon RS (2005) Learning-related fMRI activation associated with a rotational visuo-motor transformation. Brain Res Cogn Brain Res 22:373–383. 10.1016/j.cogbrainres.2004.09.007 15722208

[B30] Hadipour-Niktarash A, Lee CK, Desmond JE, Shadmehr R (2007) Impairment of retention but not acquisition of a visuomotor skill through time-dependent disruption of primary motor cortex. J Neurosci 27:13413–13419. 10.1523/JNEUROSCI.2570-07.2007 18057199 PMC6673085

[B31] Haith AM, Pakpoor J, Krakauer JW (2016) Independence of movement preparation and movement initiation. J Neurosci 36:3007–3015. 10.1523/JNEUROSCI.3245-15.2016 26961954 PMC6601759

[B32] Hamel R, Trempe M, Bernier PM (2017) Disruption of M1 activity during performance plateau impairs consolidation of motor memories. J Neurosci 37:9197–9206. 10.1523/JNEUROSCI.3916-16.2017 28821677 PMC6596746

[B33] Henriques DY, Cressman EK (2012) Visuomotor adaptation and proprioceptive recalibration. J Mot Behav 44:435–444. 10.1080/00222895.2012.659232 23237466

[B34] Herwig U, Satrapi P, Schönfeldt-Lecuona C (2003) Using the international 10-20 EEG system for positioning of transcranial magnetic stimulation. Brain Topogr 16:95–99. 10.1023/B:BRAT.0000006333.93597.9d 14977202

[B35] Homan RW, Herman J, Purdy P (1987) Cerebral location of international 10-20 system electrode placement. Electroencephalogr Clin Neurophysiol 66:376–382. 10.1016/0013-4694(87)90206-9 2435517

[B36] Inoue K, Kawashima R, Satoh K, Kinomura S, Goto R, Sugiura M, Ito M, Fukuda H (1997) Activity in the parietal area during visuomotor learning with optical rotation. Neuroreport 8:3979–3983. 10.1097/00001756-199712220-00026 9462478

[B37] Inoue K, Kawashima R, Satoh K, Kinomura S, Sugiura M, Goto R, Ito M, Fukuda H (2000) A PET study of visuomotor learning under optical rotation. Neuroimage 11:505–516. 10.1006/nimg.2000.0554 10806036

[B38] Inoue M, Kitazawa S (2018) Motor error in parietal area 5 and target error in area 7 drive distinctive adaptation in reaching. Curr Biol 28:2250–2262.e3. 10.1016/j.cub.2018.05.056 29983313

[B39] Izawa J, Shadmehr R (2011) Learning from sensory and reward prediction errors during motor adaptation. PLoS Comput Biol 7:e1002012. 10.1371/journal.pcbi.1002012 21423711 PMC3053313

[B40] Koch G, Fernandez Del Olmo M, Cheeran B, Ruge D, Schippling S, Caltagirone C, Rothwell JC (2007) Focal stimulation of the posterior parietal cortex increases the excitability of the ipsilateral motor cortex. J Neurosci 27:6815–6822. 10.1523/JNEUROSCI.0598-07.2007 17581969 PMC6672690

[B41] Koch G, Fernandez Del Olmo M, Cheeran B, Schippling S, Caltagirone C, Driver J, Rothwell JC (2008) Functional interplay between posterior parietal and ipsilateral motor cortex revealed by twin-coil transcranial magnetic stimulation during reach planning toward contralateral space. J Neurosci 28:5944–5953. 10.1523/JNEUROSCI.0957-08.2008 18524898 PMC2648507

[B42] Krakauer JW, Ghilardi MF, Mentis M, Barnes A, Veytsman M, Eidelberg D, Ghez C (2004) Differential cortical and subcortical activations in learning rotations and gains for reaching: a PET study. J Neurophysiol 91:924–933. 10.1152/jn.00675.2003 14523069

[B43] Krakauer JW, Hadjiosif AM, Xu J, Wong AL, Haith AM (2019) Motor learning. Compr Physiol 9:613–663. 10.1002/cphy.c170043 30873583

[B44] Lakens D (2013) Calculating and reporting effect sizes to facilitate cumulative science: a practical primer for t-tests and ANOVAs. Front Psychol 4:863. 10.3389/fpsyg.2013.00863 24324449 PMC3840331

[B45] Lefaucheur JP, André-Obadia N, Poulet E, Devanne H, Haffen E, Londero A, Cretin B, Leroi AM, Radtchenko A, Saba G, Thai-Van H, Litré CF, Vercueil L, Bouhassira D, Ayache SS, Farhat WH, Zouari HG, Mylius V, Nicolier M, Garcia-Larrea L (2011) [French guidelines on the use of repetitive transcranial magnetic stimulation (rTMS): safety and therapeutic indications]. Neurophysiol Clin 41:221–295. 10.1016/j.neucli.2011.10.062 22153574

[B46] Luauté J, Schwartz S, Rossetti Y, Spiridon M, Rode G, Boisson D, Vuilleumier P (2009) Dynamic changes in brain activity during prism adaptation. J Neurosci 29:169–178. 10.1523/JNEUROSCI.3054-08.2009 19129395 PMC6664918

[B47] Maris E, Oostenveld R (2007) Nonparametric statistical testing of EEG- and MEG-data. J Neurosci Methods 164:177–190. 10.1016/j.jneumeth.2007.03.024 17517438

[B48] Mazzoni P, Krakauer JW (2006) An implicit plan overrides an explicit strategy during visuomotor adaptation. J Neurosci 26:3642–3645. 10.1523/JNEUROSCI.5317-05.2006 16597717 PMC6674132

[B49] McDougle SD, Bond KM, Taylor JA (2015) Explicit and implicit processes constitute the fast and slow processes of sensorimotor learning. J Neurosci 35:9568–9579. 10.1523/JNEUROSCI.5061-14.2015 26134640 PMC4571499

[B50] Moisello C, Blanco D, Fontanesi C, Lin J, Biagioni M, Kumar P, Brys M, Loggini A, Marinelli L, Abbruzzese G, Quartarone A, Tononi G, Di Rocco A, Ghilardi MF (2015) TMS enhances retention of a motor skill in Parkinson’s disease. Brain Stimul 8:224–230. 10.1016/j.brs.2014.11.005 25533243 PMC4314317

[B51] Morehead JR, Taylor JA, Parvin D, Ivry RB (2017) Characteristics of implicit sensorimotor adaptation revealed by task-irrelevant clamped feedback. J Cogn Neurosci 29:1061–1074. 28195523 10.1162/jocn_a_01108 PMC5505262

[B52] Mutha PK, Sainburg RL, Haaland KY (2011a) Left parietal regions are critical for adaptive visuomotor control. J Neurosci 31:6972–6981. 10.1523/JNEUROSCI.6432-10.2011 21562259 PMC3107546

[B53] Mutha PK, Sainburg RL, Haaland KY (2011b) Critical neural substrates for correcting unexpected trajectory errors and learning from them. Brain 134:3647–3661. 10.1093/brain/awr275 22075071 PMC3235559

[B54] Newport R, Brown L, Husain M, Mort D, Jackson SR (2006) The role of the posterior parietal lobe in prism adaptation: failure to adapt to optical prisms in a patient with bilateral damage to posterior parietal cortex. Cortex 42:720–729. 10.1016/S0010-9452(08)70410-6 16909632

[B55] Newport R, Jackson SR (2006) Posterior parietal cortex and the dissociable components of prism adaptation. Neuropsychologia 44:2757–2765. 10.1016/j.neuropsychologia.2006.01.007 16504222

[B56] Nikooyan AA, Ahmed AA (2015) Reward feedback accelerates motor learning. J Neurophysiol 113:633–646. 10.1152/jn.00032.2014 25355957

[B57] Nitsche MA, Cohen LG, Wassermann EM, Priori A, Lang N, Antal A, Paulus W, Hummel F, Boggio PS, Fregni F, Pascual-Leone A (2008) Transcranial direct current stimulation: state of the art 2008. Brain Stimul 1:206–223. 10.1016/j.brs.2008.06.004 20633386

[B58] Okamoto M, Dan H, Sakamoto K, Takeo K, Shimizu K, Kohno S, Oda I, Isobe S, Suzuki T, Kohyama K, Dan I (2004) Three-dimensional probabilistic anatomical cranio-cerebral correlation via the international 10-20 system oriented for transcranial functional brain mapping. Neuroimage 21:99–111. 10.1016/j.neuroimage.2003.08.026 14741647

[B59] Oostenveld R, Praamstra P (2001) The five percent electrode system for high-resolution EEG and ERP measurements. Clin Neurophysiol 112:713–719. 10.1016/s1388-2457(00)00527-7 11275545

[B60] Overduin SA, Richardson AG, Bizzi E (2009) Cortical processing during dynamic motor adaptation. Adv Exp Med Biol 629:423–438. 10.1007/978-0-387-77064-2_22 19227513

[B61] Panico F, Sagliano L, Grossi D, Trojano L (2018) Bi-cephalic parietal and cerebellar direct current stimulation interferes with early error correction in prism adaptation: toward a complex view of the neural mechanisms underlying visuomotor control. Cortex 109:226–233. 10.1016/j.cortex.2018.09.020 30391877

[B62] Pelli DG (1997) The VideoToolbox software for visual psychophysics: transforming numbers into movies. Spat Vis 10:437–442. 10.1163/156856897X00366 9176953

[B63] Pisella L, Gréa H, Tilikete C, Vighetto A, Desmurget M, Rode G, Boisson D, Rossetti Y (2000) An “automatic pilot” for the hand in human posterior parietal cortex: toward reinterpreting optic ataxia. Nat Neurosci 3:729–736. 10.1038/76694 10862707

[B64] Pisella L, Michel C, Gréa H, Tilikete C, Vighetto A, Rossetti Y (2004) Preserved prism adaptation in bilateral optic ataxia: strategic versus adaptive reaction to prisms. Exp Brain Res 156:399–408. 10.1007/s00221-003-1746-4 15133651

[B65] Premoli I, Castellanos N, Rivolta D, Belardinelli P, Bajo R, Zipser C, Espenhahn S, Heidegger T, Müller-Dahlhaus F, Ziemann U (2014) TMS-EEG signatures of GABAergic neurotransmission in the human cortex. J Neurosci 34:5603–5612. 10.1523/JNEUROSCI.5089-13.2014 24741050 PMC6608220

[B66] Prime SL, Vesia M, Crawford JD (2008) Transcranial magnetic stimulation over posterior parietal cortex disrupts transsaccadic memory of multiple objects. J Neurosci 28:6938–6949. 10.1523/JNEUROSCI.0542-08.2008 18596168 PMC6670980

[B67] Richardson AG, Overduin SA, Valero-Cabré A, Padoa-Schioppa C, Pascual-Leone A, Bizzi E, Press DZ (2006) Disruption of primary motor cortex before learning impairs memory of movement dynamics. J Neurosci 26:12466–12470. 10.1523/JNEUROSCI.1139-06.2006 17135408 PMC6674906

[B68] Salomonczyk D, Cressman EK, Henriques DY (2011) Proprioceptive recalibration following prolonged training and increasing distortions in visuomotor adaptation. Neuropsychologia 49:3053–3062. 10.1016/j.neuropsychologia.2011.07.006 21787794

[B69] Salomonczyk D, Henriques DY, Cressman EK (2012) Proprioceptive recalibration in the right and left hands following abrupt visuomotor adaptation. Exp Brain Res 217:187–196. 10.1007/s00221-011-2985-4 22198532

[B70] Savoie FA, Thénault F, Whittingstall K, Bernier PM (2018) Visuomotor prediction errors modulate EEG activity over parietal cortex. Sci Rep 8:12513. 10.1038/s41598-018-30609-0 30131580 PMC6104041

[B71] Seidler RD, Noll DC, Chintalapati P (2006) Bilateral basal ganglia activation associated with sensorimotor adaptation. Exp Brain Res 175:544–555. 10.1007/s00221-006-0571-y 16794848

[B72] Seidler RD, Noll DC (2008) Neuroanatomical correlates of motor acquisition and motor transfer. J Neurophysiol 99:1836–1845. 10.1152/jn.01187.2007 18272874

[B73] Shadmehr R, Holcomb HH (1997) Neural correlates of motor memory consolidation. Science 277:821–825. 10.1126/science.277.5327.821 9242612

[B74] Shadmehr R (2018) Motor learning: a cortical system for adaptive motor control. Curr Biol 28:R793–R795. 10.1016/j.cub.2018.05.071 30040941

[B75] Simani MC, McGuire LM, Sabes PN (2007) Visual-shift adaptation is composed of separable sensory and task-dependent effects. J Neurophysiol 98:2827–2841. 10.1152/jn.00290.2007 17728389 PMC2536598

[B76] Sliwinska MW, Vitello S, Devlin JT (2014) Transcranial magnetic stimulation for investigating causal brain-behavioral relationships and their time course. J Vis Exp (89):51735. 10.3791/51735 PMC4219631 25079670

[B77] Smith MA, Ghazizadeh A, Shadmehr R (2006) Interacting adaptive processes with different timescales underlie short-term motor learning. PLoS Biol 4:e179. 10.1371/journal.pbio.0040179 16700627 PMC1463025

[B78] Stewart LM, Walsh V, Rothwell JC (2001) Motor and phosphene thresholds: a transcranial magnetic stimulation correlation study. Neuropsychologia 39:415–419. 10.1016/s0028-3932(00)00130-5 11164880

[B79] Tan H, Wade C, Brown P (2016) Post-movement beta activity in sensorimotor cortex indexes confidence in the estimations from internal models. J Neurosci 36:1516–1528. 10.1523/JNEUROSCI.3204-15.2016 26843635 PMC4737767

[B80] Tanaka H, Sejnowski TJ, Krakauer JW (2009) Adaptation to visuomotor rotation through interaction between posterior parietal and motor cortical areas. J Neurophysiol 102:2921–2932. 10.1152/jn.90834.2008 19741098 PMC2777823

[B81] Taylor JA, Ivry RB (2011) Flexible cognitive strategies during motor learning. PLoS Comput Biol 7:e1001096. 10.1371/journal.pcbi.1001096 21390266 PMC3048379

[B82] Taylor JA, Krakauer JW, Ivry RB (2014) Explicit and implicit contributions to learning in a sensorimotor adaptation task. J Neurosci 34:3023–3032. 10.1523/JNEUROSCI.3619-13.2014 24553942 PMC3931506

[B83] Torrecillos F, Alayrangues J, Kilavik BE, Malfait N (2015) Distinct modulations in sensorimotor postmovement and foreperiod β-band activities related to error salience processing and sensorimotor adaptation. J Neurosci 35:12753–12765. 10.1523/JNEUROSCI.1090-15.2015 26377464 PMC6795206

[B84] Vesia M, Monteon JA, Sergio LE, Crawford JD (2006) Hemispheric asymmetry in memory-guided pointing during single-pulse transcranial magnetic stimulation of human parietal cortex. J Neurophysiol 96:3016–3027. 10.1152/jn.00411.2006 17005619

[B85] Vesia M, Yan X, Henriques DY, Sergio LE, Crawford JD (2008) Transcranial magnetic stimulation over human dorsal-lateral posterior parietal cortex disrupts integration of hand position signals into the reach plan. J Neurophysiol 100:2005–2014. 10.1152/jn.90519.2008 18684904

[B86] Vesia M, Prime SL, Yan X, Sergio LE, Crawford JD (2010) Specificity of human parietal saccade and reach regions during transcranial magnetic stimulation. J Neurosci 30:13053–13065. 10.1523/JNEUROSCI.1644-10.2010 20881123 PMC6633525

[B87] Werner S, Schorn CF, Bock O, Theysohn N, Timmann D (2014) Neural correlates of adaptation to gradual and to sudden visuomotor distortions in humans. Exp Brain Res 232:1145–1156. 10.1007/s00221-014-3824-1 24449008

